# Using Health Impact Assessment as an Interdisciplinary Teaching Tool

**DOI:** 10.3390/ijerph14070744

**Published:** 2017-07-08

**Authors:** Melissa Chinchilla, Mariana C. Arcaya

**Affiliations:** Department of Urban Studies and Planning, Massachusetts Institute of Technology, 77 Massachusetts Ave, Cambridge, MA 02139, USA; myc@mit.edu

**Keywords:** health impact assessment, social determinants of health, public health, urban planning

## Abstract

Health Impact Assessment (HIA) courses are teaching public health and urban planning students how to assess the likely health effects of proposed policies, plans, and projects. We suggest that public health and urban planning have complimentary frameworks for training practitioners to address the living conditions that affect health. Planning perspectives emphasize practical skills for impacting community change, while public health stresses professional purpose and ethics. Frameworks from both disciplines can enhance the HIA learning experience by helping students tackle questions related to community impact, engagement, social justice, and ethics. We also propose that HIA community engagement processes can be enriched through an empathetic practice that focuses on greater personal introspection.

## 1. Introduction

Health Impact Assessment (HIA) uses scientific evidence, professional expertise, and stakeholder input to evaluate the different paths through which proposed policies, plans, and projects may affect health, and makes recommendations to maximize benefits and minimize risks [1,2,3]. Internationally, HIA may be conducted for various reasons; HIA may be mandated, serve as a decision-support instrument, be used as an advocacy tool, or constitute a community led effort to understand proposed local change [4]. In the United States HIA is generally not mandated, but is voluntarily used to analyze proposed actions through a health lens. HIA is an evidence-based assessment tool that aims to inform decision-making processes and is primarily applied to proposals in non-health sectors, such as housing, transportation, and employment, with potential to impact population health outcomes [5]. HIA consists of six stages: screening, scoping, risk assessment, report preparation, report submission, and monitoring or evaluation. These steps provide a structured way to identify proposals that will benefit from formal assessment, to set assessment boundaries, prepare reports, disseminate findings, and monitor outcomes [6]. Throughout the process, HIA emphasizes the Social Determinants of Health (SDH), which include the social and environmental factors that affect human health, as opposed to a narrow focus on medical and genetic causes [7,8]. Research identifies the SDH at the root of growing health inequalities and persistent population health concerns [9].

SDH impact public health, but they are not modifiable by public health agencies alone [10]. Addressing population health requires policy changes that are cross-sectoral. A call for “healthy urban planning” is an effort to bridge the relationship between city planning and public health by examining how community development policies impact health outcomes [11,12], but concrete tools for bridging the disciplines are lacking. HIA provides a space for public health and planning to work collaboratively, exchange expertise, and explore divergent views and approaches [13]. HIA courses are being incorporated into urban planning curriculum, primarily taught as cross-listed courses between urban planning and public health departments [14]. These courses bring together students from planning and public health disciplines, creating an environment that facilitates interaction and knowledge exchange between the two groups. Furthermore, collaboration in the classroom may translate to increased partnerships on the ground after students graduate, as future practitioners become aware of what knowledge and expertise each discipline can contribute to efforts to improve population health.

In order to give students experience with the practice of HIA, courses may include work with community-based organizations interested in using student expertise to assess the health impacts of policies and development proposals. Interactive learning through community-based projects is a staple of urban planning curriculum, and one that has been identified as having potential value in the field of public health [15]. Service learning combined with reflective practice contributes practical insights for grappling with the complexities of on-the-ground-work. However, some of the most challenging issues that may arise through service learning include questions of how to work in diverse communities, and what the potential impact is project work for people’s daily lives. These challenges encompass ethical questions of social justice, fairness, marginalization, and power, topics that public health traditions and empathetic practice may offer insights for addressing.

In this paper we suggest that HIA is a tool for bringing together urban planning and public health by jointly educating future practitioners to promote the development and advancement of policies that address population health concerns ethically, and are socially and politically actionable. While other researchers have also examined the value of HIA courses [14], we extend this conversation by exploring how HIA’s emphasis on community engagement raises questions regarding work with marginalized and vulnerable populations, and suggest that emphasizing the role of empathy in practice can help the next generation of HIA practitioners develop a more comprehensive understanding of community needs. Calling on professional HIA experience and teaching a semester long HIA course to 10 students from a mix of urban planning, public health, and environmental science backgrounds at the undergraduate, graduate, and continuing professional levels, we highlight how planning’s use of reflective practice, public health’s ethical frameworks, and the use of empathy in practice can help future practitioners tackle fundamental questions related to (1) community impact, (2) social justice and ethics, and (3) community engagement. We begin by discussing planning’s use of service learning and reflective practice as pedagogical tools for preparing students to engage with multiple stakeholders and to gain practical skills to impact systemic change. We then highlight how health perspectives on ethics and social justice can provide students with valuable guidance for addressing professional obligations and community needs. Lastly, although empathetic practice has been minimally explored in both planning and public health fields, we point to the potential value of empathy in facilitating community engagement and enabling students to develop a more nuanced view of community members’ experiences and needs.

## 2. The Importance of Hands-On Experience and Reflective Practice

Planning curriculum focuses on teaching students the skills needed to engage multiple stakeholders in creating and implementing policies and development plans [16]. It stresses the importance of getting students ready to work in the field in varying contexts and towards a number of different goals [16,17]. Preparing students for practice is partially accomplished through the use of service learning, an educational approach that combines learning objectives and community service [18]. In this section we discuss the value of planning’s use of service learning and reflection. We note that these tools are not deeply rooted in public health traditions, and suggest that community projects and reflective practice can add value to the HIA learning experience and student’s future work.

Service learning may include a wide range of projects, such as student work with community based organizations to provide technical expertise, gather stakeholder input, and/or make policy and development recommendations. Students who engage with real world concerns are provided an opportunity to negotiate dilemmas that are not easily taught in the classroom. They are positioned to do more than passively receive of information, and must actively engage with, and interpret their surroundings [19]. Service learning helps students engage with material learned in class, improve critical thinking and problem solving skills, gain professional experience, and view issues from multiple perspectives including their own, the organization’s, and community members’ [20,21,22,23]. Service learning, including internships and client projects, are incorporated in some form in most undergraduate and postgraduate planning programs [16,24].

In planning, service learning is often combined with reflective practice. Reflective practice consists of students thinking critically about what they are doing, how they are doing it, and the repercussions of their actions [25]. In 1983, Donald Schön, Professor of Urban Studies and Education, popularized reflective practice in his pivotal book “The Reflective Practitioner” in which he emphasized the importance of thinking critically about one’s work [25]. Schön [26] described reflection in two ways: (1) thinking about what you are doing while you are doing it, and (2) engaging in a conversation about experiences in order to make conceptual and practical connections. If students actively think about what they are doing when they engage in practice, they are partaking in what Schön termed “reflection-in-action.” Here a practitioner studies how a situation changes based on his or her actions, taking in feedback and learning to alter his or her approaches in real time [27]. Reflective practitioners undertake their work with self-awareness, incorporating a balance between action and reflection [28]. The process of reflecting on practice allows students to consider how they can apply tools to new situations, improve collaboration, and move from knowing what to do to knowing how to do it [29,30,31].

Service learning and reflective practice have both been identified as having potential value for public health practitioners [15,32]. Public health professionals are expected to work collaboratively with various stakeholders as well as address complex behavioral and social factors that impact community health [33]. When combined with health disparity and social justice frameworks that emphasize the SDH, health-focused service learning gives students practical experience understanding issues from multiple perspectives, increasing their sensitivity to diversity, and deepening their understanding of how structural causes shape health outcomes [20,21,22,34]. In addition, service learning creates a reciprocal relationship in which students engage with course materials through community work and communities benefit from students’ contributions, an outcome that is in line with public health’s focus on social justice [20].

The health promotion literature suggests that practice that incorporates reflection has the potential to address health inequalities emphasized under the SDH by pushing practitioners to consistently reflect on their professional values and guiding ethics [35,36,37]. Tretheway and colleagues [32] argue that if health promotion is to uphold its ethical values, critical reflection must be included as a core competency for practitioners. Ethical practice requires that practitioners engage in thoughtful, systematic analysis [38].

In planning schools, reflective practice is intentionally built into classroom instruction. Reflective practice should include ongoing class discussions and written reflection assignments, allowing students to think about challenges and progress made on client projects. Incorporating reflective practice into service projects helps students explore the tension between what they learn in the classroom and the complicated world of practice. Students can use their experiences in practice to gain insight and improve future work endeavors [31]. In this way, service learning is a tool to educate reflective practitioners.

Service learning is a way to connect students to the people and places that they will serve. Kupiec [39] notes that this type of engagement “makes students more sensitive to the environments in which they serve and to the consequences of their actions” (p.9). Through our experience teaching HIA, we find that service-learning projects can be a rewarding experience. However, engaging with community stakeholders brings up questions regarding moral reasoning, social responsibility, and the role of intercultural understanding [39,40]. Community based projects lead students to grapple with their role as impartial experts versus advocates, competing community needs, and the challenges of interacting with diverse publics. Below we outline challenges students may face as they navigate service learning in HIA, and how instructors may address these challenges by clearly defining professional goals and introducing a focus on empathetic practice.

## 3. Ethics and Professional Purpose Matter

HIA courses that allow students to engage in hands-on work highlight the complexities of applying classroom instruction to real world problems. When working with marginalized and/or vulnerable populations, students will find that these communities have multiple, and sometimes competing, needs, creating a struggle to maintain a clear focus on project goals that are limited by semester-long timeframes. Students may feel pulled to examine all problems negatively impacting the lives of community members, and instructors should anticipate that unrealistic scopes can set students up to over-commit on deliverables or stretch themselves thin, risking a failure to produce project work that is of substantial benefit to community partners. In this section, we suggest that using the HIA scoping phase to clearly focus on critical population health needs can help improve students’ experience, as well as the deliverables they produce through service learning projects. We highlight the different roles that ethics play in public health versus planning disciplines to illustrate how the fields differ in prioritizing issues, and how public health frameworks can benefit planning students as they narrow their HIA scopes.

During the HIA scoping phase, students set project boundaries by identifying aims, the population that will be impacted by the HIA, and the methods that will be utilized to conduct assessment. In this phase, students should consult with experts and community stakeholders to gather input that is likely to increase HIA utility and success. Planning students are taught to address multiple community concerns with no rigid definition of what goals are most important, which may be in tension with HIA's focus on health. Among other issues, planners give housing, transportation, employment, and environmental policy comparable weight. While all are important for a community’s health and wellbeing, HIA may require students to concentrate on a few key issues that are likely to have significant impacts on community health outcomes. This type of narrowing of focus requires an acknowledgment that the primary goal of HIA is to address health, which can be introduced to students through discussions of the fundamental differences between the goals of urban planning and public health.

A profession’s guiding principles and its justification for specific courses of action are captured within its ethical frameworks. Planning’s focus on spatial intervention demands work that is context-specific and shaped by local standards [41]. Planning stresses that all situations are different and that there is no one-size-fits-all model [42]. Planners must deal with uncertainty, multiple stakeholders, and conflicting values and goals. They need to be able to address the importance of context and adapt practice to particulars. Planning requires an emphasis on action that finds significant tension between what “is” and what “ought” to be [43]. Planners’ work is often driven by value-based judgments that presuppose some choices are better than others. As a result, planning ethics have focused on normative theories that address questions of action, with minimal attention to why specific actions should be taken [44].

Normative ethics focus on what should be done, and assume that some ethical judgments are better than others without exploring why this may be the case [45]. In contrast, meta-ethics examine how value-based judgments are made and help us understand the foundation of ethical values, including questions such as “What is justice?” [44]. Meta-ethics do not engage in questions of what ought to be done. Some planning scholars argue that meta-ethics are irrelevant to planning theory and practice [43,44]. Planning curriculum focuses on teaching students tools for practice and stresses professional ethics tied to action including “public decision-making, research, and client representation” [16]. However, students need to understand meta-ethics to analyze why they are working towards a particular outcome; meta-ethics provide a justification or underlying purpose for a chosen course of action. Winkler and Duminy [45] note, “meta-ethics cannot assist us in deciding which ethical principles are right or wrong, better or worse, or how we should plan. Rather, our use of meta-ethics allows us to begin to understand how planners (and other actors) distinguish between “better” or “worse” decision-making processes and outcomes, how they arrive at a moral judgment in the first place, how they employ ethical principles in practice, and how they justify their interventions on ethical grounds” (p. 10). Understanding the fundamental drive for one’s work can be important when faced with multiple potential outcomes. This is particularly true in the case of HIAs that deal with issues in non-health sectors that have the potential to affect stakeholders in various ways. For example, a proposed landfill may negatively affect residents’ health through environmental exposures but may also provide much needed employment for a low-income community. As a field, planning does not weigh one as more important than the other. In contrast, public health’s fundamental focus is pursuing improved health, and weighs costs and benefits in this light [46].

In contrast to planning, public health's grounding in both meta-ethical and normative frameworks is easier to trace, stemming from health research and medical practice ethics. Medical ethics emerged as a response to the moral dilemmas of medical care and human research. Early framers stressed the importance of patients’ rights to autonomy, including informed consent and the right to refuse treatment [47,48,49,50]. Physicians were expected to provide the “best care for the dignity of man in patients or research subjects” [51]. Kass [52] states that the key foundations of bioethics are relevant for public health, with the exception of its notable priority of individual autonomy, which cannot always be expected when dealing with community level issues. Public health professionals see the protection of health as a moral mandate. Emerging from within the bioethics framework, four guiding principles have been applied to the ethical analysis of healthcare problems: autonomy, beneficence, non-maleficence, and justice [53].

In contrast to medicine, which improves health at the individual level, public health addresses well-being at the population level. Public health is defined by the Institute of Medicine [54] as, “the fulfillment of society's interest in assuring the conditions in which people can be healthy” (p.40). Public health moves away from the physician-patient relationship and interfaces with various community stakeholders to improve health outcomes [55]. Public health ethics acknowledge that human rights must be weighed against communitarian concerns for population health. Systems and people are interdependent, and decisions made and actions taken must be understood from this perspective [56]. Nancy Kass [52] proposed one of the earliest ethical frameworks for public health based on rights and social justice. Kass views rights as consisting of both negative and positive rights. Negative rights are citizens’ right to noninterference; positive rights emphasize public health’s obligation to improve the public’s health and reduce social inequalities. Kass stressed the practitioner’s role in improving health, reducing health disparities, engaging in thoughtful ethical analysis, and using factual evidence to advocate for interventions. At the meta-ethical level, public health focuses on the task of improving population health as a goal. There is agreement that public health’s obligation is to prevent harm and protect health, which gives the profession firm purpose for action and serves as a spring board to develop normative ethical principles [57].

Public health aims to improve health and is also committed to focusing on the needs of the most disadvantaged [10]. Efforts to promote health equity should be seen in the context of fairness and justice [10,58]. A common definition of justice is fairness in the way that people are treated and how decisions are made [59]. Justice stresses the distribution of burdens and benefits [10], and a focus on marginalized and disadvantaged groups is seen as advancing the common good of a community [10]. If health is important because it is imperative for justice, then the field of public health is driven by a moral concern to remedy ill-health [60]. Beauchamp [61] states, “The historic dream of public health…is a dream of social justice” (p.105).

An emphasis on fairness and justice requires particular attention to the role that health plays in allowing individuals to achieve their full potential [62]. Health has a unique value for individuals, for it is fundamental for functioning in society and exercising one’s human rights [62]. A focus on social justice is what drives public health practitioners to address important health issues, including reducing inequality by targeting the SDH [10]. Using the framework of justice as health equity makes it clear for students working on HIA that their responsibility is to protect and advocate for the health of communities. While multiple concerns may be at stake when examining proposed policies and plans, health is viewed as the preeminent goal.

Early course material can be chosen to introduce students to the ethical impetus for tackling population health. An introduction to HIA should include material that explores health as a human right, how public health conceptualizes its professional mission, and gives examples of HIAs that have created healthy community change. Through reflective practice, students should be pushed to explore what ethical values are personally important for them and what underlies these beliefs. Personal reflection can occur through class discussions that ask students why they are interested in HIA, about past work experience, and future professional goals. Instructors should encourage students to make connections between HIA practice and personal beliefs and motivations.

## 4. Community Engagement and the Role of Empathy

Planning stresses the importance of action. Students are trained through hands-on work that focuses on understanding the complexities of practice. Understanding these complexities is essential in HIAs that tackle the intricacies of community development and policy change. In addition, public health’s objective of addressing health inequalities gives students a clear goal. Both planning and public health recognize the importance of community engagement, and addressing health disparities requires that practitioners assure that previously marginalized communities have a seat at the decision-making table [63]. However, both disciplines provide minimal guidance on working with diverse and marginalized populations. Given this limitation, we suggest that empathetic practice can enhance community engagement practices.

Incorporating community members’ lived experiences, needs, and preferences into HIA are key parts of assessment. Students are likely to work with communities that they are not part of, that they are not familiar with, and whose needs and experiences they may not understand. Instructors should acknowledge that these differences can create a risk of “othering,” or thinking of people or groups as being fundamentally different from oneself, which can result in exclusion and marginalization of those being “othered” [64]. Service learning provides important opportunities for students to engage with questions of diversity and can be facilitated to reduce the risk of othering. Sletto [65] suggests “service learning pedagogy should provide the necessary space and conceptual tools for students to analyze the narratives of place, self, and other…” (p.403). The importance of developing a framework for understanding oneself in relation to those served is particularly poignant given racial and ethnic disparities in health [66,67,68,69,70] and the history of service learning models, which have been criticized for frequently pairing White, middle-class students with low-income communities of color [71].

Courses that use community engagement as a learning tool must address issues of racism, privilege, and power. How students interact with and treat vulnerable populations is important for promoting interactions and policies that are inclusive and respectful [72]. In our experience, community engagement is one of the most notable challenges of service learning because it raises questions of representation, difference, understanding, and community agency.

Students working with local communities in HIA engagement processes can find themselves in the midst of personal and emotional testimony about how policy and development plans are affecting community members’ physical and mental well-being. These emotionally charged encounters impact how students interpret community needs, community power, and agency. While sympathy to residents’ stories is a natural reaction, instructors should focus on empathy by encouraging an understanding of individuals’ experiences and recognizing community agency. For outsiders who do not come from communities like those they are working with, it is easy to overestimate the centrality of HIA projects to local organizing activities overall. A distorted perspective on how isolated projects relate to ongoing, community-led action to solve community issues creates at least two problems. First, it discounts community members’ roles as agents of change and, secondly, it creates distress among those engaged in service learning. Implementing an empathetic approach in HIA courses can help educators preempt these and other challenges associated with community engagement with diverse and vulnerable populations.

Empathy represents an active sharing of what others are experiencing; sympathy is an expression of concern or sorrow. Empathy enables one to develop a sense of understanding of someone’s experience and acknowledge a person’s agency in addressing problems at hand. In contrast, sympathy consists of judgment of a person’s needs [73]. Research shows that we are able to understand other people’s viewpoints from how they display emotion [74], and the development of empathy can increase the accuracy with which we understand what others aim to convey [75]. Urban planning literature makes little if any mention of the importance of empathy in practitioners’ work [76,77,78]. Forester [79] notes, “to work effectively, planners must be able to respond to others’ ideas and to their passions: their fears, suspicions, distrust, anger, and so on. But this is emotional work that planners are poorly trained to do” (p. 256). As proposed by Bronfenbrenner and Morris [80], individuals are embedded in an ecological system where multiple environments are interdependent and contribute to one another. However, planning has largely concentrated on mezzo and macro level issues, focusing on systems and policy frameworks that impact social and physical environments with minimal attention paid to the emotional and ethical challenges that practitioners face when working with vulnerable populations [78,81]. Empathy is often thought of at the individual level. As a field, public health has tried to move away from an exclusive focus on the individual level, which is seen as reductionist [82]. Empathy has primarily been viewed as relevant to healthcare and patient-physician interactions. However, medical frameworks that incorporate empathy may be a useful starting point for understanding why an empathetic approach is important in community engagement.

Medical frameworks based on work with patients at the individual level can offer students a lens for understanding the importance of interpersonal relationships in health advocacy. Within the medical field, the patient–physician relationship is an important piece of recovery [83]. Halpern [84] states that empathy makes patients more willing to share their symptoms and concerns, which results in more accurate diagnosis and care; it encourages patients to participate in their recovery, allowing them to exercise their autonomy and increase their self-efficacy; and an empathetic approach enables therapeutic interactions that are beneficial for the patient and physician. Physicians are more effective in their work, and experience greater professional satisfaction, when they engage in an empathetic practice [85].

Healthcare acknowledges the importance of patient-centered approaches, including paying attention to patients’ views on care [86,87,88,89]. Clinical empathy includes three components: (1) understanding a patient’s situation and how the patients feels about it; (2) communicating this understanding and checking with the patient to assess accuracy of interpretation; and (3) using this understanding to help the patient address the issue at hand [90]. Halpern [84] suggests that clinical empathy is based on the emotional resonance between the patient and healthcare professional. Formal teaching approaches have focused on teaching empathy as either a behavioral skill that can be taught or as an attitude that must be personally tended to [91]. Medicine proposes that empathy can be taught both in the classroom and through interactive learning approaches [92,93,94,95]. Kasl and Yorks [96] highlight three educational principles valued in developing empathy in adult education: “(1) Significant learning is grounded in the learner’s life experience, (2) significant learning integrates multiple ways of knowing, and (3) dialogue contributes markedly to significant learning” (p. 4).

Medical perspectives on empathy can help students understand why interpersonal relationships and recognizing "different ways of knowing" are important in community interactions. Developing empathy requires getting to know others and acknowledging people’s multi-dimensionality. Students should be encouraged to interact with community members as much as is welcome by the communities they serve. Students must engage stakeholders in project decisions, and should attend community meetings and interact with stakeholders through in-class visits. When stakeholders feel comfortable, not unduly burdened, and otherwise eager to do so, community members sharing personal and emotional testimony about how proposals may affect their lives can create meaningful, rather than distressing, experiences for students. Community engagement should be an opportunity for students to learn about community members’ individual experiences; personal stories provide useful information for HIA reports, and also help personalize population health issues. By learning about others’ experiences, we see the world through another’s perspective, which is key for developing empathy.

Relationship building is essential for community engagement, and can help students navigate the HIA process, including potential hurdles. For example, HIAs rely heavily on objective, scientific evidence that, sometimes, may not support community claims. Students who develop an empathetic practice and want to assure a community’s wellbeing may struggle with how to handle apparent discrepancies between the scholarly evidence base and community voices. Research may not support community claims for two reasons: (1) data sets or scientific studies relevant to specific community concerns have not been gathered or published, resulting in a situation where there is no evidence to support community claims; and (2) robust evidence exists that contradicts a community’s claims. Students should be able to distinguish between these two situations, and explain them to both stakeholders and outside audiences. In interpreting apparent conflicts between the scientific evidence base and community claims, students should keep in mind that the two are different sources of insight, and that practitioners often must triangulate among multiple sources of knowledge to reach conclusions using HIA. When weighing evidence, students should consider whether results are generalizable to the population that they are working with, if constructs used are valid, and whether study findings hold true under current local conditions. In cases where strong scientific evidence is squarely at odds with community claims, empathy can push students to thoughtfully explain research findings to stakeholders, and to be careful in their HIA reports to recognize that a community’s claims can reflect important aspects of lived experiences even if evidence does not support health impact assertions.

## 5. Recommendations for Teaching HIA

This section presents suggestions for integrating the previously discussed concepts and themes into HIA courses. First, introductions to HIA should be grounded in the history of public health, including the aims of the profession, and how and why HIA was developed. The introduction should also address how inequality, poverty, and racism affect health, and why public health practitioners frame their work within a social justice lens. Students should be introduced to evidence on how the SDH impact people’s daily lives, what structural changes are needed to improve population health, and the power and limitations of health lenses and HIA as advocacy tools.

Throughout the course, students should be required to meet with their community partners and seek community input into the HIA process. We recommend that students visit local partners in the community, attend community meetings, and keep partners involved in the HIA process through regular updates. In our course, we also facilitated presentations where students presented their work and gathered stakeholder feedback. Due to logistical constraints students presented in the classroom. However, holding presentations in the local community, when possible, may encourage greater community ownership of the HIA.

Lastly, instructors should incorporate reflective practice throughout the course. Students should reflect on HIA progress, successes, and failures through in class discussion and written assignments. Students should be asked what can be improved in the HIA process and how these improvements can be achieved. We found that having an outside facilitator lead reflection can be helpful. In our experience, the outside facilitator was able to take the place of a neutral observer, bringing a new perspective to class dynamics, and encouraging students to thoroughly explain experiences. The facilitator, a Professor of Practice from Massachusetts Institute of Technology’s Department of Urban Planning, was asked to come towards the end of the class. The facilitator led a class exercise that inquired about students’ experiences throughout the course. Students were asked what events were noteworthy, and probed how they felt when events occurred and what they learned from them. The exercise provided an opportunity for an in depth introspective conversation.

## 6. Conclusions

HIA provides an opportunity for interdisciplinary collaboration, bringing together planning and public health students, allowing them to exchange expertise, and learning what each field can bring to HIA practice. Through our experience teaching HIA, we find that coursework and student service learning projects can offer future practitioners valuable tools and perspectives for practice. Using frameworks emphasized in urban planning, specifically service learning and reflective practice, students have the opportunity to gain practical skills. Public health ethics provide a strong foundational basis in which students are able to ground their actions. Lastly, empathy is an emotional skill that can help students engage community stakeholders in more open and productive ways by allowing students to acknowledge the complexities of experience and the power of community agency.

HIA student learning projects can allow students to engage in reflective practice, learn by doing, and develop relationships with local communities. Work completed for community partners shapes student learning and can have meaningful impacts in the daily lives of local citizens. Hands-on experience, combined with the opportunity to critically examine work undertaken, provides invaluable learning experiences for students and prepares them to address obstacles they will encounter in their professional endeavors. As Baum [30] states, “practice is learned by practicing” (p.21). In addition, community projects may direct benefits to local communities [97], and studies show that students that engage with hands-on work outside of the classroom remember what they learned for a longer period of time [98].

HIA courses have the opportunity to bring multiple stakeholders to the table. HIA should aim to involve all stakeholders that may be affected by the decision under consideration. Increasing stakeholder involvement makes the decision process more transparent, and those involved understand potential consequences more clearly [1]. Community engagement processes enrich HIA, and also provide important learning experiences for students who will go on to work with local communities. Students engaging with diverse communities will also likely tackle questions related to diversity, power, and agency. These interactions, especially those with diverse communities, require the development of empathetic practice. Community engagement is emotional work. Emotions influence how we process information and the actions that we decide to take. Emotions are a fundamental component of our experiences and a site of knowledge [99].

Through the HIA course experience, students can come to see HIA as one potential tool to advocate for community change and positively impact individuals’ lives. Students learn that health can be viewed as a fundamental right, and a value that can be used to move policy forward. They may also begin to acknowledge the ways in which health affects myriad aspects of individuals’ lives that planners focus on; students learn how issues are interconnected, and that health promotion can serve as a motivation for community and policy change. Educators are encouraged to view HIA courses as an opportunity to train interdisciplinary practitioners to consider how they can make their professional contributions inclusive, ethical, and actionable in the service of population health promotion.

While we propose several ways that the HIA learning experience can be enhanced, we acknowledge that there are limitations and challenges to implementation. Through our experience, we find that semester long courses may result in logistical challenges. First, both students and community members have competing demands. Difficulty coordinating schedules may limit the number of interactions between students and community stakeholders. In addition, the HIA process often takes longer than a semester to complete. Although students may turn in a complete HIA report, dissemination and evaluation of the report may occur when the course is over or even after the students’ have graduated. Advocacy activities, such as testifying in public hearings and educating others about the HIA, may also occur after the course. In all these cases, community stakeholders will bear the brunt of the burden as students have moved on to other endeavors. In addition, developing empathetic practice is not a semester long project, but a continuous learning experience for both educators and students.

## References

[1] Kemm J. (2001). Health impact assessment: A tool for healthy public policy. Health Promot. Int..

[2] Council N.R. (2011). Improving Health in the United States: The Role of Health Impact Assessment.

[3] Mindell J.S., Boltong A., Forde I. (2008). A review of health impact assessment frameworks. Public Health.

[4] Harris-Roxas B., Harris E. (2011). Differing forms, differing purposes: A typology of health impact assessment. Environ. Impact Assess. Rev..

[5] Lock K. (2000). Health impact assessment. BMJ.

[6] Mindell J., Ison E., Joffe M. (2003). A glossary for health impact assessment. J. Epidemiol. Community Health.

[7] Marmot M., Friel S., Bell R., Houweling T.A., Taylor S., Commission on Social Determinants of Health (2008). Closing the gap in a generation: Health equity through action on the social determinants of health. Lancet.

[8] Kawachi I., Berkman L. (2000). Social cohesion, social capital, and health. Soc. Epidemiol..

[9] Marmot M. (2005). Social determinants of health inequalities. Lancet.

[10] Gostin L.O., Powers M. (2006). What does social justice require for the public’s health? Public health ethics and policy imperatives. Health Aff..

[11] Corburn J. (2009). Toward the Healthy City: People, Places, and the Politics of Urban Planning.

[12] Duhl L.J., Sanchez A.K., Organization W.H. (1999). Healthy Cities and the City Planning Process: A Background Document on Links between Health and Urban Planning.

[13] Corburn J. (2007). Reconnecting with our roots: American urban planning and public health in the twenty-first century. Urban Aff. Rev..

[14] Pollack K.M., Dannenberg A.L., Botchwey N.D., Stone C.L., Seto E. (2015). Developing a model curriculum for a university course in health impact assessment in the USA. Impact Assess. Proj. Apprais..

[15] Rooks R.N., Rael C.T. (2013). Enhancing curriculum through service learning in the social determinants of health course. J. Scholarsh. Teach. Learn..

[16] Planning Accreditation Board PAB Accreditation Standards. http://www.planningaccreditationboard.org/index.php?id=41.

[17] Association of Collegiate Schools of Planning (ACSP) (2017). What Is Planning?.

[18] Levesque-Bristol C., Knapp T.D., Fisher B.J. (2011). The effectiveness of service-learning: It’s not always what you think. J. Exp. Educ..

[19] Kirschner P., Van Vilsteren P., Hummel H., Wigman M. (1997). The design of a study environment for acquiring academic and professional competence. Stud. High. Educ..

[20] Cashman S.B., Seifer S.D. (2008). Service-learning: An integral part of undergraduate public health. Am. J. Prev. Med..

[21] Peterson B.A., Yockey J., Larsen P., Twidwell D., Jorgensen K. (2006). Service-learning projects: Meeting community needs. Home Health Care Manag. Pract..

[22] Reeb R.N. (2010). Service-Learning in Community Action Research: Introduction to the Special Section. Am. J. Community Psychol..

[23] Freestone R., Thompson S., Williams P. (2006). Student experiences of work-based learning in planning education. J. Plan. Educ. Res..

[24] Myers D. (1997). Anchor points for planning’s identification. J. Plan. Educ. Res..

[25] Schön D.A. (1984). The Reflective Practitioner: How Professionals Think in Action.

[26] Schön D.A. (1987). Educating the Reflective Practitioner: Toward a New Design for Teaching and Learning in the Professions.

[27] Schon D.A., DeSanctis V. (2011). The Reflective Practitioner: How Professionals Think in Action. J. Cont. High. Educ..

[28] Kinsella E.A. (2001). Reflections on Reflective Practice. Can. J. Occup. Ther..

[29] Baum H.S. (1997). Teaching Practice. J. Plan. Educ. Res..

[30] Connor-Greene P.A. (1999). Bridging the gap between the ivory tower and the real world: Problem based learning and service learning.

[31] Brooks K.R., Nocks B.C., Farris J.T., Cunningham M.G. (2002). Teaching for practice: Implementing a process to integrate work experience in an MCRP curriculum. J. Plan. Educ. Res..

[32] Tretheway R., Taylor J., O’Hara L., Percival N. (2016). A missing ethical competency? A review of critical reflection in health promotion. Health Promot. J. Aust..

[33] Bernheim R.G., Nieburg P., Bonnie R.J. (2007). Ethics and the Practice of Public Health.

[34] Loewenson K.M., Hunt R.J. (2011). Transforming attitudes of nursing students: Evaluating a service-learning experience. J. Nurs. Educ..

[35] Parker E., Gould T., Fleming M. (2007). Ethics in health promotion-reflections in practice. Health Promot. J. Aust..

[36] Massé R., Williams-Jones B. (2012). Ethical dilemmas in health promotion practice. Health Promot. Can..

[37] Fleming P. (2007). Enhancing the empowerment agenda in health promotion through reflective practice. Reflect. Pract..

[38] Carter S.M. (2014). Health promotion: An ethical analysis. Health Promot. J. Aust..

[39] Kupiec T.Y. (1993). Rethinking Tradition: Integrating Service with Academic Study on College Campuses.

[40] Denise P.S., Harris I.M. (1989). Experiential Education for Community Development. Contributions to the Study of Education, Number 31.

[41] Merriman P., Jones M., Olsson G., Sheppard E., Thrift N., Tuan Y.-F. (2012). Space and spatiality in theory. Dialog. Hum. Geogr..

[42] Healey P. (2012). The universal and the contingent: Some reflections on the transnational flow of planning ideas and practices. Plan. Theory.

[43] Campbell H. (2012). Planning ethics 1 and rediscovering the idea of planning 2. Plan. Theory.

[44] Jacobs J. (2008). Dimensions of Moral Theory: An. Introduction to Metaethics and Moral Psychology.

[45] Winkler T., Duminy J. (2016). Planning to change the world? Questioning the normative ethics of planning theories 1. Plan. Theory.

[46] Hendler S. (1995). Planning Ethics: A Reader in Planning Theory, Practice, and Education.

[47] Callahan D. (1973). Bioethics as a Discipline. Hastings Cent. Stud..

[48] Callahan D. (1984). Autonomy: A Moral Good, Not a Moral Obsession. Hastings Cent. Rep..

[49] Steinbock B. (1996). Liberty, responsibility, and the common good. Hastings Cent. Rep..

[50] Edmund D. Pellegrino, for the Patient’s Good: The Restoration of Beneficence in Health Care-PhilPapers. https://philpapers.org/rec/PELFTP.

[51] Ramsey P. (1973). The nature of medical ethics. Proceedings of the National Conference on the Teaching of Medical Ethics.

[52] Kass N.E. (2001). An ethics framework for public health. Am. J. Public Health.

[53] Beauchamp T.L., Childress J.F. (2001). Principles of Biomedical Ethics.

[54] Institute of Medicine (US) Committee for the Study of the Future of Public Health (1988). The Future of Public Health.

[55] Childress J.F., Faden R.R., Gaare R.D., Gostin L.O., Kahn J., Bonnie R.J., Kass N.E., Mastroianni A.C., Moreno J.D., Nieburg P. (2002). Public health ethics: Mapping the terrain. J. Law Med. Ethics.

[56] A Code of Ethics for Public Health AJPH Vol. 92, Issue 7. http://ajph.aphapublications.org/doi/abs/10.2105/AJPH.92.7.1057.

[57] Lee L.M. (2012). Public health ethics theory: Review and path to convergence. J. Law Med. Ethics.

[58] Krieger N., Birn A.-E. (1998). A vision of social justice as the foundation of public health: Commemorating 150 years of the spirit of 1848. Am. J. Public Health.

[59] Rawls J. (2009). A Theory of Justice.

[60] Venkatapuram S., Marmot M. (2009). Epidemiology and social justice in light of social determinants of health research. Bioethics.

[61] Beauchamp D.E. (1976). Public health as social justice. Inquiry.

[62] Sen A. (2009). The Idea of Justice.

[63] Tesh S.N. (2000). Uncertain Hazards: Environmental Activists and Scientific Proof.

[64] Weis L. (1995). Identity formation and the processes of “othering”: Unraveling sexual threads. Educ. Found..

[65] Sletto B. (2010). Educating reflective practitioners: Learning to embrace the unexpected through service learning. J. Plan. Educ. Res..

[66] Byrd W.M., Clayton L.A. (2000–2002). An American Health Dilemma.

[67] Williams D.R., Collins C. (2001). Racial residential segregation: A fundamental cause of racial disparities in health. Public Health Rep..

[68] Ba Bashir S.A. (2002). Home is where the harm is: Inadequate housing as a public health crisis. Am. J. Public Health.

[69] Cummins S.K., Jackson R.J. (2001). The built environment and children’s health. Pediatr. Clin. N. Am..

[70] Kawachi I. (1999). Social capital and community effects on population and individual health. Ann. N. Y. Acad. Sci..

[71] Taboada A. (2011). Privilege, Power, and Public Health Programs: A Student Perspective on Deconstructing Institutional Racism in Community Service Learning. J. Public Health Manag. Pract..

[72] Daiski I. (2007). Perspectives of homeless people on their health and health needs priorities. J. Adv. Nurs..

[73] Clark A. (2010). Empathy and Sympathy: Therapeutic Distinctions in Counseling. J. Ment. Health Couns..

[74] Hochschild A.R. (1983). The Managed Heart : Commercialization of Human Feeling.

[75] Perry J. (2003). Knowledge, Possibility, and Consciousness.

[76] Umemoto K. (2001). Walking in another’s shoes—Epistemological challenges in participatory planning. J. Plan. Educ. Res..

[77] Ferreira A. (2013). Emotions in planning practice: A critical review and a suggestion for future developments based on mindfulness. Town Plan. Rev..

[78] Fischer F. (2009). Policy deliberation: Confronting subjectivity and emotional expression. Crit. Policy Stud..

[79] Forester J. (1996). 12 Argument, Power, and Passion in Planning Practice. Explor. Plan. Theory.

[80] Bronfenbrenner U., Morris P. (1998). The ecology of developmental processes. Theorectical Models of Human Development.

[81] Hoch C. (2006). Emotions and Planning. Plan. Theory Pract..

[82] Pearce N. (1996). Traditional Epidemiology, Modern Epidemiology, and Public Health. Am. J. Public Health.

[83] Di Blasi Z., Kleijnen J. (2003). Context Effects: Powerful Therapies or Methodological Bias?. Eval. Health Prof..

[84] Halpern J. (2001). From Detached Concern to Empathy. [Electronic Resource]: Humanizing Medical Practice.

[85] Larson E.B., Yao X. (2005). Clinical empathy as emotional labor in the patient-physician relationship. JAMA.

[86] Tim U. (1999). Understanding The Consultation: Evidence, Theory and Practice.

[87] Gore J., Ogden J. (1998). Developing, validating and consolidating the doctor-patient relationship: The patients’ views of a dynamic process. Br. J. Gen. Pract..

[88] Cromarty I. (1996). What do patients think about during their consultations? A qualitative study. Br. J. Gen. Pract..

[89] Stewart M. (2001). Towards a global definition of patient centred care: The patient should be the judge of patient centred care. Br. Med. J..

[90] Mercer S.W., Reynolds W.J. (2002). Empathy and quality of care. Br. J. Gen. Pract..

[91] Shapiro J. (2002). How do physicians teach empathy in the primary care setting?. Acad. Med..

[92] Winefield H.R., Chur-Hansen A. (2000). Evaluating the outcome of communication skill teaching for entry-level medical students: Does knowledge of empathy increase?. Med. Educ..

[93] Kramer D., Ber R., Moore M. (1989). Increasing Empathy among Medical-Students. Med. Educ..

[94] Branch W.T., Kern D., Haidet P., Weissmann P., Gracey C.F., Mitchell G., Inui T. (2001). Teaching the Human Dimensions of Care in Clinical Settings. JAMA.

[95] Kolb D.A. (2014). Experiential Learning: Experience as the Source of Learning and Development.

[96] Kasl E., Yorks L. (2016). Do I Really Know You? Do You Really Know Me? Empathy Amid Diversity in Differing Learning Contexts. Adult Educ. Q..

[97] Roakes S.L., Norris-Tirrell D. (2000). Community Service Learning in Planning Education: A Framework for Course Development. J. Plan. Educ. Res..

[98] Kinsley C.W. (1994). What is community service learning?. Vital Speeches Day.

[99] Jones O. (2005). An emotional ecology of memory, self and landscape. Emotional Geographies.

